# Co-exposure to polystyrene nanoparticles and tetrabromobisphenol A induces genotoxicity and interferes with intestinal barrier differentiation

**DOI:** 10.1007/s10565-026-10228-6

**Published:** 2026-07-10

**Authors:** Patricia Soto-Bielicka, Soledad Sanz-Alférez, M. José Hazen, Paloma Fernández Freire, Ana Peropadre

**Affiliations:** Biology Department, Faculty of Sciences, Autonomous University of Madrid, 2 Darwin Street, Lab A115, Biology Building, 28049 Madrid, Spain

**Keywords:** Tetrabromobisphenol A, Polystyrene nanoparticles, Intestinal barrier, Co-exposure, Genotoxicity

## Abstract

Tetrabromobisphenol A (TBBPA) and polystyrene nanoparticles (PSNPs) are contaminants of emerging concern frequently detected in environmental matrices, with dietary exposure representing a major route. Despite their co-occurrence, data on combined toxicity remain limited. This study evaluated the effects of TBBPA and PSNPs individual and combined exposure on human intestinal barrier integrity, differentiation and function using differentiated Caco-2 cells and Caco-2/HT29-MTX co-cultures as validated *in vitro* models of increasing complexity. Cells were exposed to TBBPA (1–150 µM) and PSNPs (0.1–50 µg/mL) individually or in combination, either for 24 h in fully differentiated monolayers or continuously during the 21-day differentiation period. In differentiated Caco-2 cells, combined exposure altered cytoskeletal organization, with PSNPs being internalized and accumulating as large intracellular aggregates that persisted for at least 144 h post-exposure. Ultrastructural analysis revealed microvilli alterations and multilamellar bodies formation, although tight junction architecture remained intact and cellular adhesion structures recovered progressively following contaminant removal, suggesting retained barrier repair capacity. During the differentiation period, TBBPA was the primary cytotoxic agent and PSNPs alone showed no cytotoxicity at the concentrations tested, as confirmed by benchmark dose analysis, which yielded low relative potency factor values for PSNPs across all tested combinations. Genotoxicity assessment revealed oxidative DNA damage in selected TBBPA and PSNPs combinations using the FPG-modified comet assay, with gene expression analysis indicating activation of DNA damage response and oxidative stress pathways. Co-exposure modulated the expression of intestinal differentiation markers, including ALPI, suggesting interference with epithelial maturation processes. The complexity of the dose–response relationships observed underscores the importance of comprehensive dose-range testing and the use of physiologically relevant differentiated models in the risk assessment of contaminant mixtures.

**Graphical Abstract:**

Graphical headlights

• TBBPA is the main contributor to cytotoxicity during co-exposure with PSNPs.

• PSNPs persist within intestinal epithelial cells up to 144 h after exposure.

• Co-exposure induces oxidative stress and DNA damage in intestinal epithelial cells.

• Combined exposure alters intestinal epithelial differentiation and barrier maturation.

**Figure:**
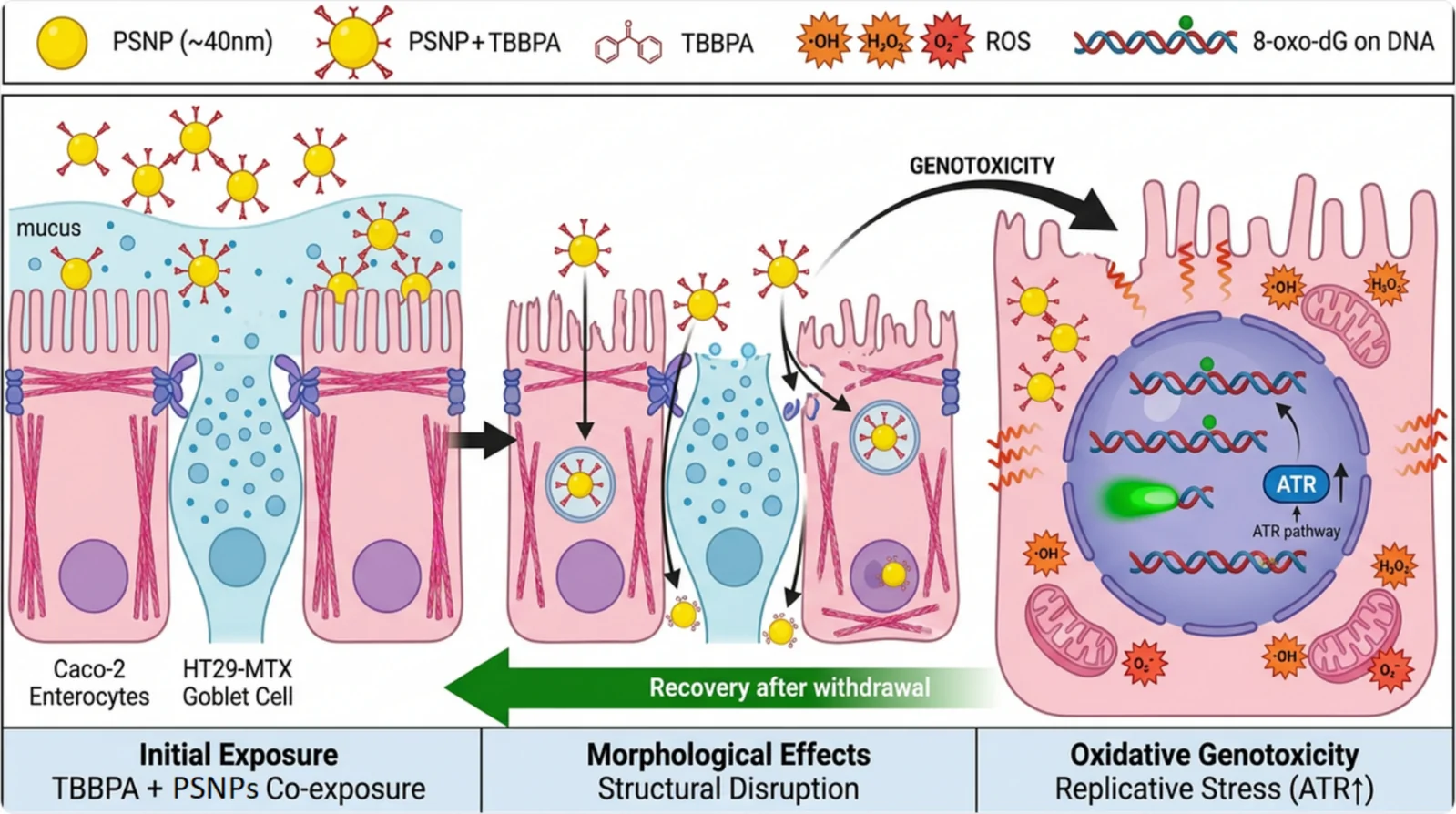

**Supplementary Information:**

The online version contains supplementary material available at https://doi.org/10.1007/s10565-026-10228-6.

## Introduction

The growing worldwide manufacture of chemical substances is a significant contributor to environmental contamination. The One Health framework has paid attention to this issue (Wang et al. 2024), highlighting the importance of developing strategies to mitigate emerging risks to both human and ecosystem health. Contaminants of emerging concern (CEC) is a relevant category of pollutants that deserves particular attention. They can be defined as substances whose occurrence, fate, and toxicity are not yet fully understood but may cause significant risks (Sauvé and Desrosiers 2014). Among them, polystyrene nanoparticles (PSNPs) and tetrabromobisphenol A (TBBPA) are relevant representatives of the plastic-derived nanoparticles and brominated flame retardants groups, respectively (Kik et al. 2020; Zhou et al. 2020). They can interact with biological systems at physiological levels (Zhou et al. 2020; Schröter and Ventura 2022), and have been associated with high environmental persistence, as PSNPs may remain in the environment for hundreds of years (Corsi et al. 2020), while TBBPA has been classified as a persistent organic pollutant with documented bioaccumulation potential (Miao et al. 2023). Aquatic organisms, including fish and invertebrates, can easily uptake PSNPs, causing morphological changes and cytotoxic effects *in vitro*, such as apoptotic features and transcriptional disruption (Li et al. 2022). TBBPA is widely present across environmental and biological matrices and is able to perturb cellular metabolism and gene expression, modifying metabolic and physiological pathways in mammalian models, including effects on immune and neuronal differentiation (Tribondeau et al. 2022; Liao et al. 2025).

Their simultaneous presence in the environment has been reported (Okeke et al. 2024), with PSNPs detected at 0.07—0.73 μg/L in surface waters (Li et al. 2022) and TBBPA at ng/L levels in freshwater systems (Wang et al. 2020), and both have been detected in human biomonitoring studies (Miao et al. 2023). Risk assessment demands toxicological characterization based not only on individual compounds, as mixture toxicity may result in additive, synergistic, or antagonistic effects that cannot be easily predicted from single-compound studies (Bopp et al. 2019). In this regard, both TBBPA and PSNPs can be found in different environmental matrices, but diet has been identified as one of the main exposure routes. For TBBPA, fish and seafood represent the primary dietary source, with concentrations reaching up to 13 µg/kg wet weight in fish liver (Schrenk et al. 2024). Based on neurotoxicity data, EFSA has established a TDI of 0.7 µg/kg body weight per day for this compound (Schrenk et al. 2024). For PSNPs, quantitative data in food matrices remain scarce, though seafood consumption is considered a relevant exposure route (Kik et al. 2020). Nevertheless, there is only limited information regarding their potential combined effects (Chen et al. 2023; Zhao et al. 2023; Soto-Bielicka et al. 2023, 2024; Liang et al. 2024; Dayana et al. 2025). Thus, the lack of mixture toxicity data represents a critical requirement that needs to be addressed.

Despite the generated concern, the mechanisms of toxic action of these compounds remains only partially understood, increasing the uncertainty regarding their health impacts. There is evidence suggesting that both compounds can interfere with fundamental cellular processes, including oxidative stress response, as demonstrated across aquatic organisms, mammalian cell lines, and rodent models (Zhang et al. 2019; Rubio et al. 2020; Liang et al. 2021; Sun et al. 2023) and inflammatory processes in both cellular and animal experimental systems (Xu et al. 2023; Bridgeman et al. 2025). The variety of experimental models and exposure conditions might be behind the lack of consensus regarding this topic. Nevertheless, the suspected pathways point to cellular stress responses that are relevant for maintaining intestinal barrier function, thus potentially increasing systemic exposure to luminal toxicants. This fact is further strengthened by the potential of PSNPs to act as a vehicle for other substances (Souza et al. 2022). This way, the potential consequences of combined exposure on intestinal structure and function stand out as a relevant research issue.

Our research group has previously addressed the combined toxicity of PSNPs and TBBPA in two complementary *in vitro* systems. In fish cell lines, individual and combined exposures revealed detrimental effects for both compounds, providing early evidence of mixture concern in aquatic vertebrate models (Soto-Bielicka et al. 2023). A subsequent study using undifferentiated human intestinal Caco-2 cells and a Caco-2/HT29-MTX co-culture system demonstrated that, under subcytotoxic conditions, TBBPA was the predominant driver of combined effects, inducing ROS generation, mitochondrial membrane potential disturbances, and DNA strand breaks, with PSNPs further modulating the expression of oxidative stress- and DNA damage-related genes (Soto-Bielicka et al. 2024). Notably, the two intestinal models exhibited differential susceptibility to mixture treatments, underscoring the relevance of model complexity. However, these studies did not address the consequences of combined exposure on Caco-2/HT29-MTX co-culture differentiation pathways or on fully intestinal barrier-like differentiated systems, which constitute the primary focus of the present work.

In this context, the present study aims to evaluate the effects of TBBPA and PSNPs combined exposure, using the colorectal human-derived Caco-2 cell line and its co-culture with HT29-MTX goblet cells. Differentiated Caco-2 cells are widely accepted as a validated model for intestinal toxicity assessment, expressing key transporters, enzymes, and tight junction proteins characteristic of human enterocytes (Ding et al. 2021). The incorporation of HT29-MTX cells into co-culture provides a mucus layer that more faithfully replicates biological properties of the intestinal barrier (Berger et al. 2017; Kleiveland 2015; Walczak et al. 2015). These complementary models allow controlled investigation of intestinal toxicity under conditions of increasing physiological complexity. We hypothesized that co-exposure to PSNPs and TBBPA can interfere with relevant parameters of the intestinal barrier, including barrier integrity, DNA damage, oxidative stress and differentiation pathways, with these effects being modulated by PSNPs concentration and intracellular retention.

## Materials and methods

### Cell cultures and treatments

The human intestinal cell lines Caco-2 (ATCC HTB-37; Virginia, USA) and HT29-MTX (ECACC 15121711; Salisbury, UK) were grown in 75 cm^2^ flasks (BioLite, Fisher Scientific, Madrid, Spain) in a HERAcell 150i incubator (Thermo Fisher, Karlsruhe, Germany), at 37 °C, 5% CO_2_ humified atmosphere, using complete culture medium (CCM) which consisted of high glucose Dulbecco’s Modified Eagle’s Medium (DMEM), with sodium pyruvate (#SH30243.01, Cytiva HyClone Laboratories, Marlborough, USA), 1% penicillin/streptomycin (#SV30010, Cytiva HyClone Laboratories, Marlborough, USA), 1% non-essential aminoacids (#11140050) and 10% foetal bovine serum (FBS) (#A5256701), both from Gibco (Life Technologies, New York, USA). Cells were subcultured when reaching a 70%—80% confluence, approximately twice per week, with sterile phosphate buffer saline (PBS) (#SH30028.03) and TrypLE Express Enzime 1X (#12605028), both from HyClone (Cytiva HyClone Laboratories, Marlborough, USA). To evaluate the morphology of the cell cultures, a phase-contrast inverted microscope Leica Dmi1 was used (Leica Microsystems, Wetzlar, Germany). All experiments were performed using cells between passages 45 and 47.

Both TBBPA (#330396, CAS 79–94–7; Sigma Aldrich, Munich, Germany) and carboxylated PSNPs (#11590066, Invitrogen, Fisher Scientific, Madrid, Spain) working concentrations were prepared in CCM. Concentrations were selected based on our previous work in undifferentiated intestinal models (Soto-Bielicka et al. 2024) and are intended for hazard identification purposes; they exceed current dietary exposure estimates and should not be interpreted as direct representations of human exposure scenarios. From a 0.1 M stock of TBBPA in dimethyl sulfoxide (Sigma Aldrich, Munich, Germany) we obtained a range of concentrations from 1 to 150 µM (0.54–81.6 µg/mL). Regarding PSNPs, the stock of 10 mg/mL in MilliQ water was used, after 30 min continuous sonication in an ultrasonic bath at room temperature (RT), to freshly prepare the highest 50 µg/mL concentration, which was further diluted to obtain all the working suspensions (0.1–50 µg/mL). These PSNPs have a nominal size of 0.04 µm and were previously characterized by dynamic light scattering (DLS) and transmission electron microscopy (TEM) as reported in Soto-Bielicka et al. (2024). When necessary, fluorescently labelled PSNPs (fPSNPs; yellow-green, 505_ex_/515_em_ nm; #F8795, Invitrogen, Fisher Scientific, Madrid, Spain) with the same physico-chemical characteristics were employed. Working suspensions of fPSNPs were prepared following the same procedure previously described for the non-fluorescent PSNPs.

A schematic overview of the experimental workflow employed in this study is provided in Supp. Figure 1 to guide the reader through the different cell models, exposure conditions, and endpoints described in the following sections.

### Evaluation of the intestinal barrier integrity

Caco-2 cells were seeded at 1.8 × 10^5^ cells/mL (8 × 10^4^ cells/cm^2^) on polycarbonate (PC) transwell inserts, (1.12 cm^2^, 0.4 µm pore size; #3401 Corning™, Fisher Scientific, Madrid, Spain) and allowed to completely differentiate for 21 days, changing their medium 2 or 3 times a week. Cells were differentiated using an asymmetric serum protocol modified from Ferruzza et al. (2012), where the apical medium was switched to serum-free conditions on Day 7 post-seeding to ensure complete monolayer confluence and prevent cell detaching, resembling more closely the physiological conditions (Hiebl et al. 2020). Differentiated (DF) cells were subsequently treated and further process as required.

#### Transepithelial electrical resistance (TEER) measurements

To monitor the differentiation state of the cells on transwell inserts and the establishment of the intestinal barrier, TEER was measured using an EVOM3 epithelial Volt/Ohm meter (World Precision Instruments, Florida, USA) every few days, starting on day 5 after the seeding, following the manufacturer's instructions, measuring the resistance of the transwell inserts. To ensure that the values obtained reflected the monolayer formation itself rather than the inherent resistance of the inserts, a blank insert without cells and treated as the control was also measured, and its resistance subtracted prior to TEER calculations. TEER measures were performed before changing the medium, including that of the blank insert, and final TEER values were calculated by multiplying the measured resistance (Ω) by the surface area of the insert (cm^2^).

#### Confocal laser scanning microscopy (CLSM)

For immunofluorescence procedures, transwell inserts were fixed with 4% paraformaldehyde (PFA) for 10 min at RT, washed with PBS, permeabilized for 10 min with 0.5% (w/v) Triton X-100, and blocked with 1% (w/v) bovine serum albumin (BSA) in PBS for 1 h. Anti E-cadherin mouse monoclonal antibody (#610,181, BD Biosciences, Madrid, Spain; 1:100 dilution) was incubated for 1 h at 37 °C in a humid chamber. Afterwards, cells were washed with PBS and likewise incubated with a secondary antibody, either Alexa 647 or Alexa 488 conjugated goat anti-mouse IgG (#A-31571 and #A-11001 respectively, Invitrogen, Fisher Scientific, Madrid, Spain; 1:500 dilution) for 1 h at 37 °C. Simultaneously, cells were incubated with Alexa 546 phalloidin (#A22283, Invitrogen, Fisher Scientific, Madrid, Spain), to stain actin microfilaments. Lastly, after nuclei counterstaining with 4′,6-diamidino-2-phenylindole (DAPI; #D1306, Invitrogen, Fisher Scientific, Madrid, Spain) for 3 min at RT and final washing in PBS, the membrane was cut from the insert and mounted on a glass slide using Vectashield plus antifade mounting medium (#H-1900–10, Vector labs, Palex Medical, Barcelona, Spain). Confocal images were captured at the “Centro de Biología Molecular Severo Ochoa” SMOC facilities, on an inverted Nikon A1R + Eclipse Ti-E microscope (Nikon Instruments Inc., New York, USA), using a 60x/1.4 Plan-Apo immersion objective (Nikon MRD01605). Laser excitation was performed using a 405 (DAPI), 488 (Alexa 488), 514 (fPSNPs) or 640 (Alexa 647) nm diode. Between 10 and 20 planes along the Z axis (Z-step: 1–2 µm) were obtained for each field, covering the full depth of the *in vitro* intestinal barrier (approximately 15–20 µm). The images were obtained with NIS Elements 5.30.05 software (Nikon) and further processed using FiJi software (Schindelin et al. 2012).

#### Transmission electron microscopy (TEM)

Cells grown on transwell inserts and treated as required, were fixed with 4% PFA/2% glutaraldehyde on 0.1 M phosphate buffer pH 7.4, 2 h at RT, washed 3 times with 0.1 M phosphate buffer pH 7.4, and post-fixated with a mixture of 1% osmium tetroxide/potassium ferrocyanide on doubled-distilled water for 1 h at 4 °C. After 3 washes with doubled-distilled water, 0.1% tannic acid on 0.1 M phosphate buffer pH 7.4 was added and incubated 1 min. Then 3 washes, the first one with 0.1 M phosphate buffer pH 7.4, and the last two with doubled-distilled water, 5 min each at RT, were conducted. This post-fixation ends with adding 2% uranyl acetate 1 h in the dark at RT and washing it with doubled-distilled water 3 times. The final steps involve progressive dehydration with ethanol and infiltration of the inserts with EPON epoxy resin. Then, transversal slices of 70 nm were made using a Leica Ultracut UCT ultramicrotome (Leica, Viena, Austria); the sections were collected on slot grids of Cu/Pd coated in Formvar and stained with 2% uranyl acetate for 7 first and lastly, 3 min with 2% Reynolds’ lead citrate.

The images were captured with a transmission electron microscopy (TEM) at the “Centro de Biología Molecular Severo Ochoa” (CBMSO) facilities on a JEM1400 Flash microscope (Jeol, Tokyo, Japan) equipped with a CMOS Oneview camera (Gatan, Pleasantville, USA). All the procedures, except the first fixation step, were performed in the CBMSO facilities. For image analysis, FiJi software (Schindelin et al. 2012) was used.

### Evaluation of the intestinal barrier differentiation process

Co-cultures were started using a 9:1 Caco-2:HT29-MTX ratio, which adequately resembles the goblet cell proportion in the human small intestine under physiological in vivo conditions (Chen et al. 2010; Karam 1999). Cells were seeded on flat transparent 96-well for cytotoxicity or 6-well plates for genotoxicity and real time RT-qPCR (#130188 and #130184, BioLite, Thermo Fisher Scientific, Massachusetts, USA) at 2 × 10^5^ cells/mL. The cells were treated during their differentiation process for up to 21 days with the compounds of interest, renewing the treatments with freshly prepared solutions twice or thrice per week. Afterwards, the cells were processed for the different experimental approaches.

#### Cytotoxicity assays

To assess the potential cytotoxicity of the compounds, two complementary viability endpoints were used: Alamar Blue (AB) to evaluate the metabolic activity, and carboxyfluorescein diacetate acetoxymethyl ester (CFDA-AM) to determine the integrity of the plasma membrane. This evaluation was performed on the same multiwell plate, a procedure previously described by Schirmer et al. (1997) and adapted to our experimental setting (Soto-Bielicka et al. 2023, 2024). Briefly, cells were incubated in DMEM without phenol red containing 5% (v/v) AB (#DAL1100, Invitrogen, Fisher Scientific, Madrid, Spain) and 4 µM CFDA-AM (#C1354, Invitrogen, Fisher Scientific, Madrid, Spain) for 30 min. Fluorescence was measured at 530/590 nm and 485/528 nm for AB and CFDA-AM respectively on a TECAN Infinite 200 PRO M Plex (Tecan Iberica Instrumentación SL, Barcelona, Spain). Background fluorescence was subtracted before normalizing the fluorescence values to the control cells. In addition, to account for nanoplastic interference, blank wells (interference blanks) were treated under the same experimental conditions as cell-containing wells and processed in parallel. Fluorescence values from these interference blanks were subtracted from the corresponding wells, yielding the values associated with the effects of the compounds or mixtures.

#### Genotoxicity evaluation

DNA damage was evaluated using the standard alkaline comet assay for the determination of strand breaks (single and double) and abasic sites, and a modified version using the formamidopyrimidine DNA glycosylase (FPG) enzyme, to determine the oxidation product 8-oxo-7, 8-dihydroguanine. Unless specifically stated, the experimental settings, reagents and solutions follow those indicated by Huarte et al. (2021). Cells were trypsinized and embedded in 22 mm^2^ gels of 0.7% low-melting agarose (LMA) disposed on agarose-precoated microscope slides (2 gels per slide), immersed in lysis buffer, washed 3 times in cold PBS and incubated at 37 °C, either with 1 U FPG (New England Biolabs, Massachusetts, USA) in appropriate buffer (NEBuffer™ 1, New England Biolabs, Massachusetts, USA), only with the buffer itself, or maintained in lysis buffer (not incubated with FPG or enzyme buffer). After rinsing the slides in cold PBS to neutralize the lysis buffer and remove the enzyme or its buffer from the slides, and 30 min unwinding, electrophoresis was performed (30 min, 0.73 V/cm). Lastly, slides were rinsed in cold PBS, washed with distilled water to remove excess PBS and prevent salt crystallization, dehydrated and nucleoids stained with GelRed (#41001, Biotium, California, USA). 10–15 field images were captured on an Olympus BX-61 microscope (Olympus, Tokyo, Japan) equipped with a DP70 camera (Olympus) and finally processed with the free CometScore 2.0 software (TriTek corp., Virginia, USA), considering the % of tail DNA of, at least, 50 nucleoids per gel. To determine the FPG sensitive sites, the mean value of %DNA in tail of gels incubated only with the enzyme buffer were subtracted from those obtained with the same treatments incubated with FPG. Appropriate positive controls, 250 µM KBrO_3_ 24 h and/or 0.1 M H_2_O_2_ 10 min, were included in all the experiments.

#### Real time RT-qPCR

To evaluate the effects of these compounds and their mixtures on DNA integrity, oxidative metabolism, and the differentiation process itself, the relative expression of different gene sets was assessed. After treatments, the cells were trypsinized and RNA was extracted using the RNeasy Mini Kit (#74106, Quiagen, Venlo, Netherlands) according to the manufacturer’s instructions. Once its quality was determined using a Nanodrop (ThermoFisher Scientific, Massachusetts, USA), the gene expression analysis was performed by real-time RT-qPCR, on the “Parque Científico de Madrid” Genomic Unit facilities, using 18 S and CycloB as housekeeping genes and the 2^-ΔΔCt method. The primers used are listed in Supp. Table 1.

To evaluate DNA damage, the expression of four DNA damage-related genes, *ATM* (mutated ataxia telangiectasia), *ATR* (ataxia telangiectasia-related protein and Rad3), *PARP1* (Poly-(ADP Ribose) Polymerase 1) and *OGG1* (8-oxoguanine DNA glycosylase 1) was determined. For oxidative metabolism, the expression of oxidative stress response–related genes was evaluated, including *GSTP1* (glutathione S-transferase pi 1), *NRF2* (nuclear erythroid factor-like 2) and *SOD2* (superoxide dismutase 2). Finally, the differentiation-related genes *ABCB1* (ATP Binding Cassette Subfamily B Member 1/P-glycoprotein), *ALPI* (intestinal alkaline phosphatase) and *TJP1* (tight junction protein/ZO-1) were assessed.

### Statistical analysis

Data plots and all statistical analysis were performed with GraphPad Prism 10.4 (GraphPad Software, San Diego, USA). A significance level of α = 0.05 was applied throughout. Homogeneity of variances was assessed using the Brown-Forsythe test prior to ANOVA.

Cytotoxicity data from individual compound exposures were analyzed by one-way ANOVA. Where variances were unequal, Dunnett's T3 *post-hoc* test was applied for pairwise comparisons against control conditions. For combined exposure cytotoxicity, a nested one-way ANOVA was used, with individual wells nested within biological experiments (n = 3), followed by Šídák-adjusted pairwise comparisons among predefined groups of interest.

Comet assay was analyzed by nested one-way ANOVA, with gels nested within biological experiments (n = 2–3 per condition, two technical replicates per experiment), followed by Šídák-adjusted pairwise comparisons. Individual nucleoid values were included for visualization purposes only and were not considered as statistical units.

RT-qPCR data were analyzed on ΔCt values, which approximate a normal distribution by construction as log2-transformed measurements (Livak & Schmittgen 2001; Schmittgen & Livak 2008), using one-way ANOVA followed by Šídák-adjusted pairwise comparisons among predefined groups of interest. Where variances were unequal, Welch's ANOVA with Games-Howell post-hoc test was used. Differences in differentiation-related gene expression between UD and fully DF cells were assessed by Welch's t-test, applied due to unequal variances between groups.

### Benchmark dose modelling

For the modelling of the dose-responses exerted by the individual and combined treatments, as well as the calculation of the Benchmark Dose (BMD), the software package PROAST version 70.3 for R (4.2.0) was used. This software package was developed by the Dutch National Institute for Public Health and the Environment (RIVM). A Benchmark Response (BMR) of 5% change relative to the control was established as the Critical Effect Size (CES = 0.05).

For individual compounds, the Critical Effect Dose (CED) was estimated along with its lower (BMDL) and upper (BMDU) confidence bounds. PSNPs did not show individual cytotoxic effects and therefore no CED could be derived for this compound.

For mixtures, a Concentration-Addition (CA) model was applied. TBBPA was selected as the reference compound, and the Relative Potency Factor (RPF) of PSNPs relative to TBBPA was calculated. Model fitting was performed automatically by PROAST with model selection based on the Akaike Information Criterion (AIC); the model yielding the lowest AIC was selected. Goodness of fit was assessed by log-likelihood ratio test (p > 0.05, indicating no significant deviation from the fitted model).

## Results

### The TBBPA-PSNPs combinations interferes with the integrity of the intestinal barrier, especially with high PSNPs concentrations

**Fig. 1:**
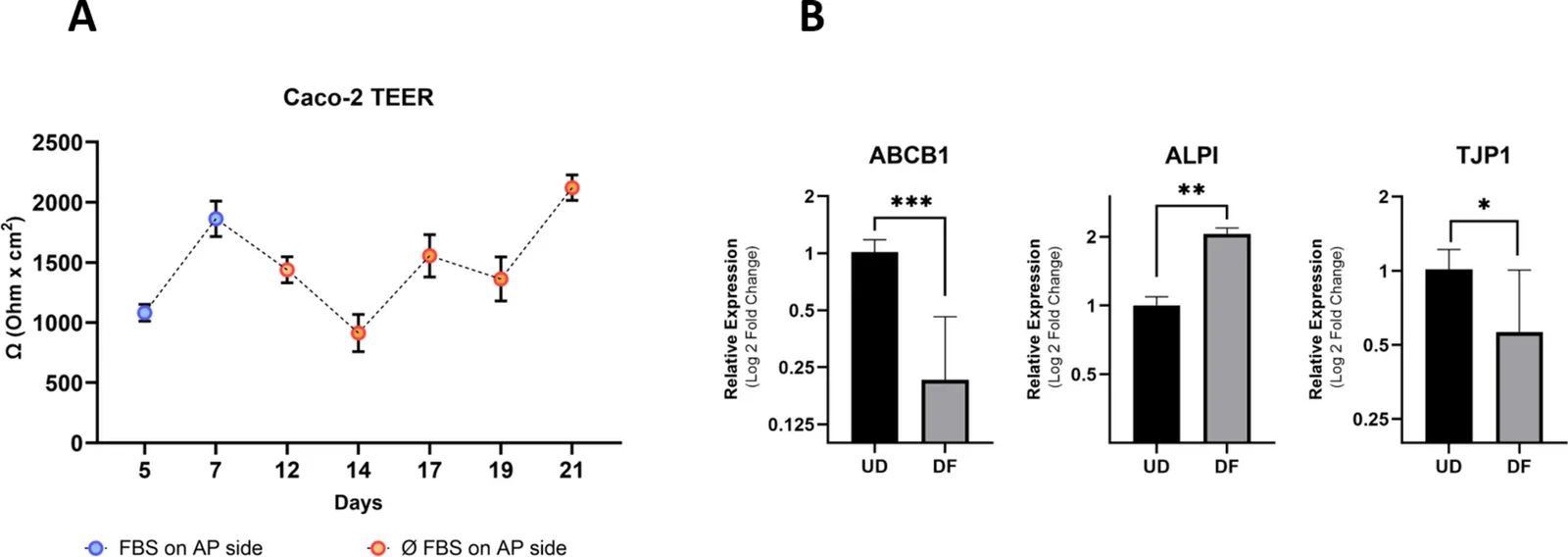
**A** TEER measurements during the differentiation process. Caco-2 cells were grown on transwell inserts for 21 days, either in complete medium (days 1–7) or in serum-free medium (from day 7). A cell-free insert with the same medium regime was used to measure baseline resistance. Data are presented as mean ± SD of 11 inserts cultured in a 12-well plate (n = 1; 11 technical replicates). AP, apical compartment. **B **Expression of selected genes involved in the differentiation process of Caco-2 cells. Bar charts showing the mean relative expression ± SD of the selected genes comparing undifferentiated (UD) and 21 days-fully differentiated (DF) untreated Caco-2 cells grown on 6-well plates (n = 3 independent experiments with 2 technical replicates per condition). Asterisks indicate statistically significant differences (p < 0.05 (*), p < 0.01 (**) or p < 0.001 (***)) determined by Welch’s t-test

To confirm proper Caco-2 cell differentiation and the formation of an intact barrier in transwell inserts prior to treatment, TEER was measured throughout the differentiation process (Fig. 1A). We detected an initial fast increase until day 7, when complete medium was replaced with serum-free medium, followed by a reduction in TEER values. After day 14, TEER progressively recovered, reaching maximum levels on day 21 day. In parallel, we monitored the differentiation process in flat multiwell plates by comparing the relative expression of differentiation-related genes between undifferentiated (UD) and differentiated (DF) Caco-2 cells (Fig. 1B). The changes detected in the three genes evaluated (ABCB1, ALPI and TJP1) showed statistically significant differences. Expression of ABCB1 and TJP1 genes decreased compared to undifferentiated cells, whereas ALPI was the only gene upregulated in differentiated Caco-2 cells (Fig. 1B).

**Fig. 2:**
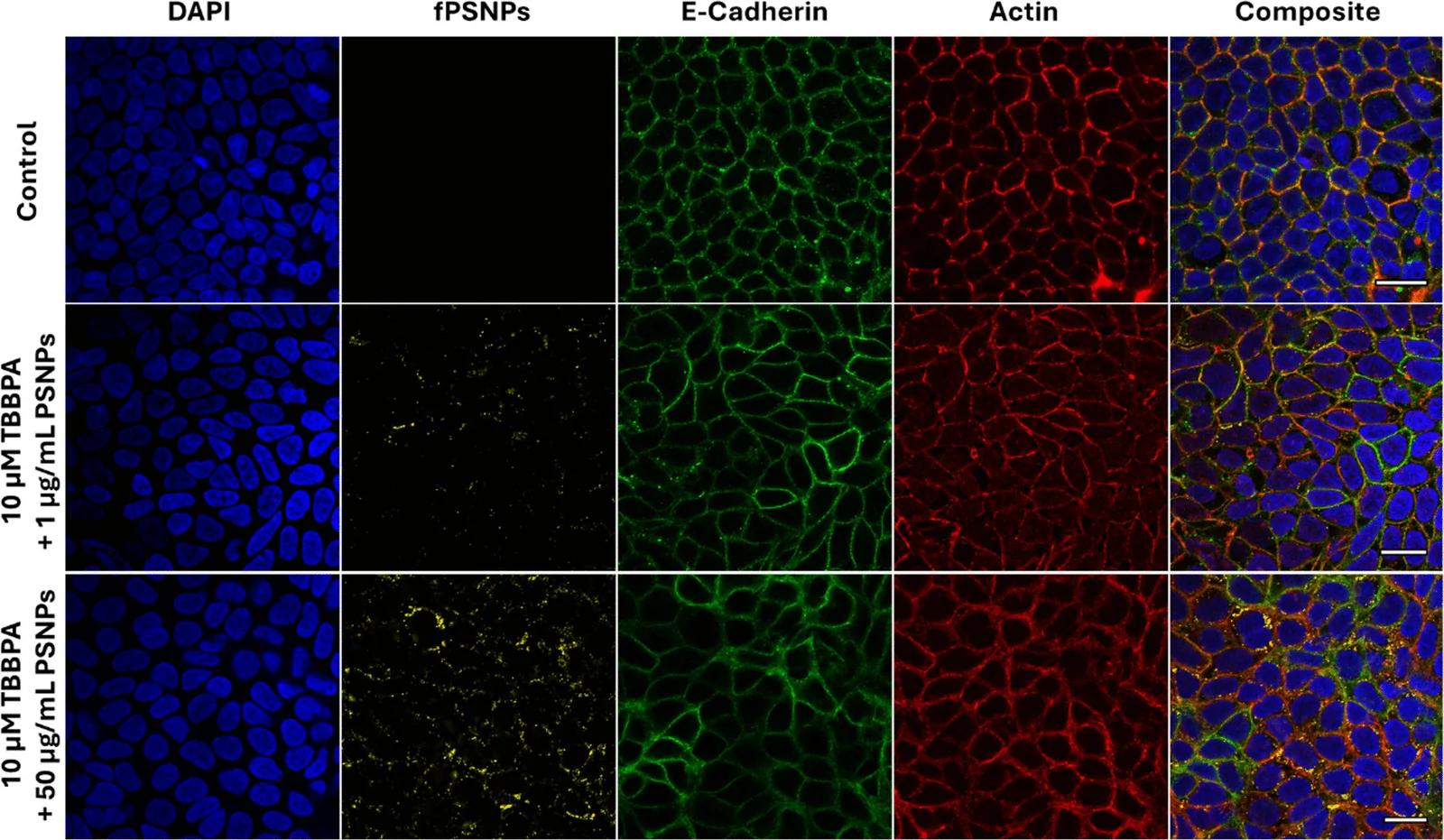
Effects on the Caco-2 monolayer integrity induced by TBBPA and fPSNPs after 24 h of exposure. Representative confocal Z-projections of Caco-2 cells differentiated on transwell inserts and exposed for 24 h to selected treatments. fPSPNs are marked in yellow, E-Cadherin in green, F-actin in red and nuclei in blue. Scale bar: 20 µm

As a follow up to our previous studies on the relevance of TBBPA-PSNPs co-exposure in undifferentiated (UD) Caco-2 (Soto-Bielicka et al. 2024), we evaluated the effects of these mixtures on the morphology and integrity of fully differentiated (DF) Caco-2 cells using fluorescent PSNPs (fPSNPs). First, DF Caco-2 monolayers were exposed to selected TBBPA and fPSNPs combinations for 24 h and analyzed by confocal microscopy. As shown in Fig. 2, fPSNPs were internalized and accumulated in large intracellular aggregates. In both combination treatments, the actin cytoskeleton and E-cadherin immunostaining, normally presenting as a sharp, well-defined signal at cell–cell contact sites, appeared broadened and less precisely localized at the junctional membrane, suggesting a reorganization of adherent junction architecture. These effects were more pronounced in cells exposed to the highest combination tested (10 µM TBBPA +50 µg/mL fPSNPs). Accordingly, a decreasing trend in TEER values was observed for the combination treatments, although this did not reach statistical significance (Supp. Figure 2).

**Fig. 3:**
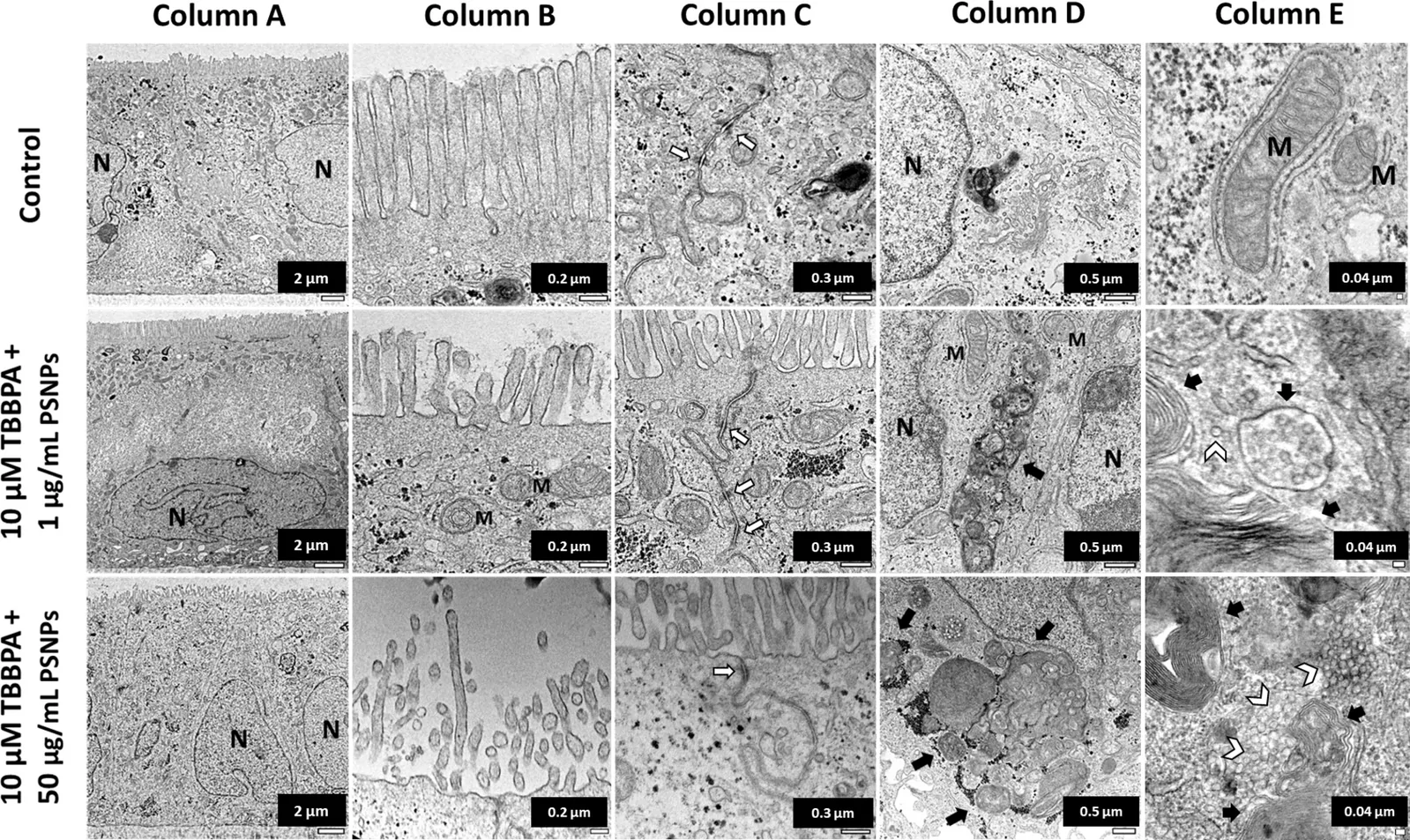
TEM images of fully differentiated Caco-2 cells grown on transwell inserts after 24 h of exposure to different TBBPA and PSNPs combinations.** Column A:** Overview showing a continuous epithelial monolayer. **Column B:** Detail of the apical microvilli brush border under different treatments. **Column C:** Detail of intercellular junctions, with electron-dense desmosomes indicated by white arrows. **Column D:** Multilamellar and multivesicular bodies in the cytoplasm, indicated by black arrows. **Column E:** PSNPs located in the cell cytoplasm, near multilamellar or multivesicular bodies, indicated by white arrow heads. N, cell nucleus; M, mitochondria

To further assess the effects of these mixtures on the intestinal barrier integrity, we examined the ultrastructure of DF Caco-2 cells in transversal sections of transwell inserts by TEM. Figure 3 shows that the general cellular architecture of the intestinal barrier remained stable after the different 24 h treatments (Fig. 3, column A). Nevertheless, microvilli abundance changed, and the brush border was damaged, especially in 10 µM TBBPA +50 µg/mL PSNPs (Fig. 3, column B). On the other hand, no noticeable alterations were observed in cell–cell interactions, including interdigitations, tight junctions, adherent junctions and desmosomes (Fig. 3, column C). Interestingly, TBBPA-PSNPs conditions showed numerous multilamellar and multivesicular bodies near which PSNPs are localized (Fig. 3, columns D and E), consistent with the intracellular aggregates previously observed by confocal microscopy.

### fPSNPs remain in large intracellular vesicular compartments even after several days

**Fig. 4:**
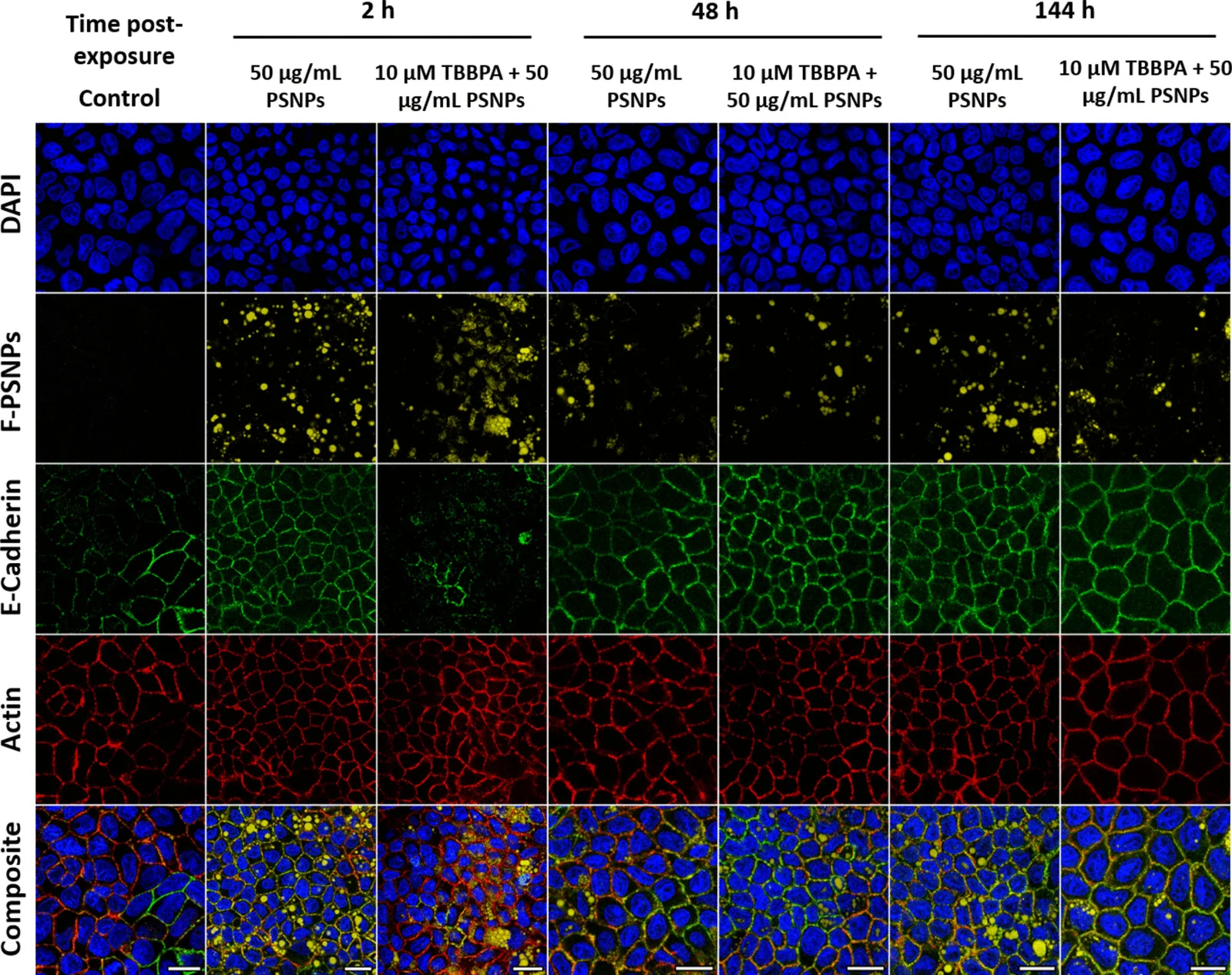
Pulse-chase assay to evaluate intracellular retention of fPSNPs. Caco-2 cells differentiated on transwell inserts were exposed for 24 h to the selected treatments and fixed at 2, 48 and 144 h after treatment removal and recovery in fresh culture medium, to evaluate fPSNPs retention or clearance over time. Representative confocal Z-projections are shown, with fPSNPs in yellow, E-cadherin in green, F-actin in red, and nuclei in blue. Scale bar: 20 µm

Given that the PSNPs were internalized by the intestinal barrier, we investigated whether cells could eliminate them at the same rate with a pulse-chase assay (Fig. 4). Thus, we examined the presence of fPSNPs inside cells at 2, 48, and 144 h post-exposure, comparing the 10 µM TBBPA +50 µg/mL fPSNPs mixture with the individual 50 µg/mL fPSNPs condition. Their presence in the treatments correlates with a certain blur of the fPSNPs signal throughout the pulse-chase experiment. Remarkably, our study shows that fPSNPs remain inside the Caco-2 cells even after 144 h post-exposure and show a fast tendency to accumulate in large intracellular vesicles. As previously observed, the 10 µM TBBPA +50 µg/mL fPSNPs mixture broadened the sharp E-cadherin and actin microfilaments pattern, a feature not detected with the individual exposure to fPSNPs. These effects were still visible after 2 h post-exposure, although the integrity of the cellular adhesion structures tends to recover over the following days. No other relevant modifications could be detected between the exposure of fPSNPs individually and their combination with TBBPA along the post-exposure period.

### Individual exposure to TBBPA during the differentiation process of human intestinal cells exerts cytotoxic effects, whereas PSNPs do not

**Fig. 5:**
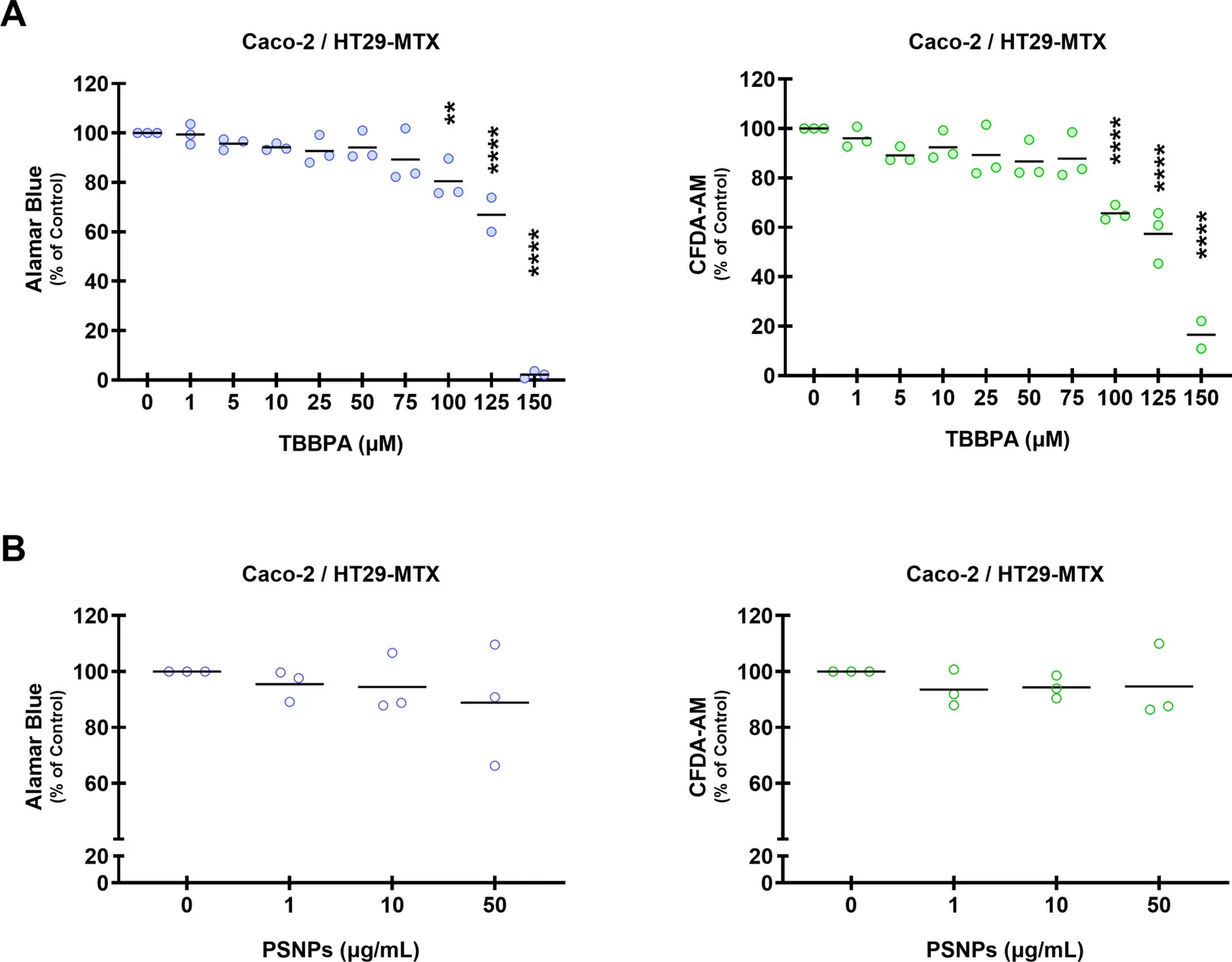
Individual cytotoxicity of Caco-2/HT29-MTX co-cultures after continuous exposure during the differentiation process to TBBPA **A **or PSNPs **B**. Scatter plots of the metabolic activity (Alamar blue, AB) and plasma membrane integrity (CFDA-AM), expressed as percentage of the control. Dots represent individual biological experiments, and the horizontal line indicates their mean (n = 3, with 3–6 technical replicates each). Asterisks indicate statistically significant differences compared to the vehicle control (** p < 0.01; **** p < 0.0001) after one-way ANOVA with Dunnett's T3 *post-hoc* test

We next evaluated the cytotoxicity of continuous individual exposure to TBBPA or PSNPs during the 21-differentiation period of Caco-2 (Supp. Figure 3) or Caco-2/HT29-MTX cell cultures (Fig. 5). Regarding the co-culture system, metabolic activity (AB) and plasma membrane integrity (CFDA-AM) showed that only TBBPA concentrations ≥ 100 µM reduced cell viability, presenting statistically significant differences. In the case of PSNPs, no significant cytotoxicity was observed with either AB or CFDA-AM assays (Fig. 5B). Consequently, relevant toxicological values calculated for TBBPA led to higher BMDs for both endpoints when the co-culture system was employed (Supp. Table 2).

### TBBPA is the primary driver of cytotoxicity in combined exposures with PSNPs after 21-day co-culture differentiation

**Fig. 6:**
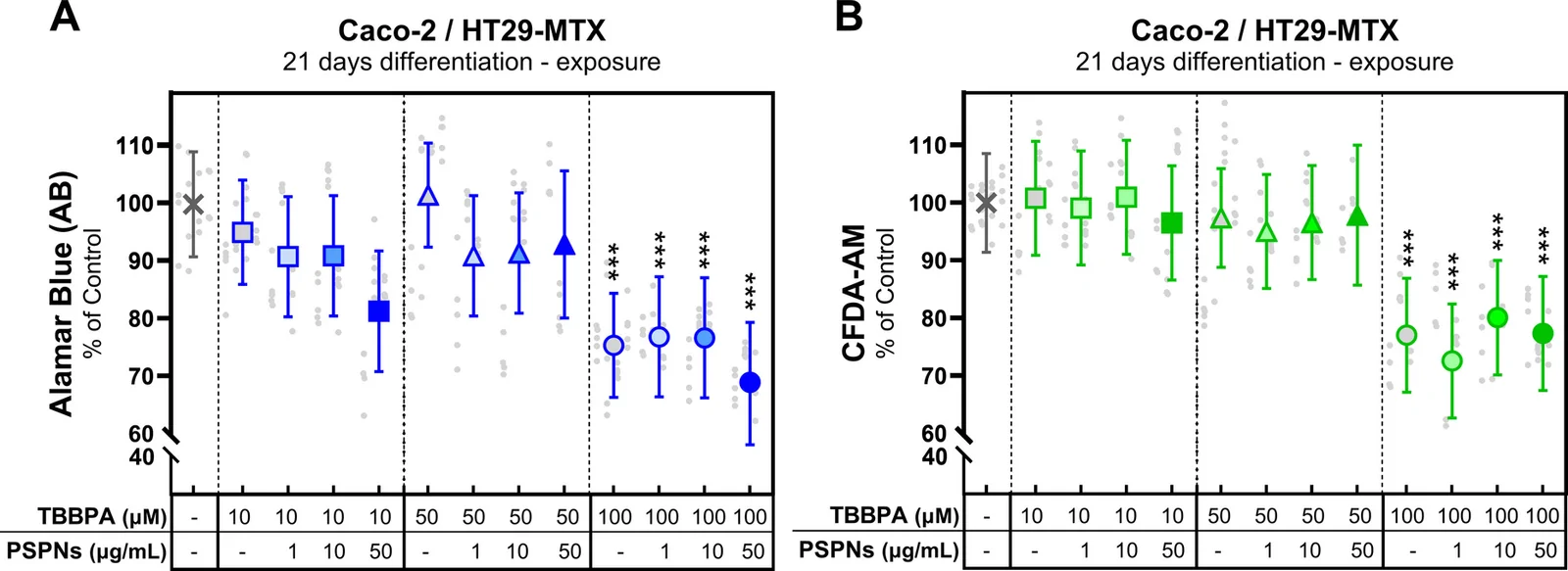
Cytotoxicity of the combined exposure of TBBPA-PSNPs during the differentiation of Caco-2/HT29-MTX co-cultures. Results show the effect of selected TBBPA concentrations combined with increasing PSNPs concentrations after 21 days of exposure on metabolic activity (AB assay) and membrane integrity (CFDA-AM assay). Grey dots represent individual wells (technical replicates) nested within each of the n = 3 independent biological experiments. Symbols and error bars indicate the mean ±95% confidence interval of independent biological experiments. Statistical comparisons were performed using nested one-way ANOVA, with wells nested within biological experiments, followed by Šídák-adjusted pairwise comparisons (α = 0.05) among predefined groups of interest

Afterwards, we determined the potential interactions of different TBBPA-PSNPs combinations on the cytotoxicity of the co-culture differentiation. We selected three TBBPA concentrations based on their individual cytotoxic effects (10, 50 and 100 µM), and the three PSNPs conditions previously employed. The results (Fig. 6) depict an interesting modification in AB behavior. At 10 and 50 µM TBBPA, combinations with increasing PSNPs concentrations showed a trend towards reduced metabolic activity compared to the corresponding individual TBBPA treatments, although these differences did not reach statistical significance. On the other hand, exposure to 100 µM TBBPA, both alone and in all combinations with PSNPs, significantly reduced AB and CFDA-AM values compared to the control, while the addition of PSNPs did not significantly alter the cytotoxic effect of TBBPA at this concentration. Overall, these results shows that cell viability tend to decrease as the PSNPs concentration increase.

The role of TBBPA as the major determinant of cytotoxicity is further supported by BMD analysis (Supp. Table 3 and Supp. Figure 4), performed using PROAST, which yielded the critical effective dose (CED) for TBBPA, and the relative potency factor (RPF) of PSNPs in relation to TBBPA in the tested combinations. The low RPF values obtained for PSNPs confirm that the predominant contributor to the cytotoxic effects of the mixtures is TBBPA, with PSNPs exerting little influence on the overall toxicity.

### Genotoxicity and DNA damage are detected after the exposure of TBBPA and PSNPs combinations

**Fig. 7:**
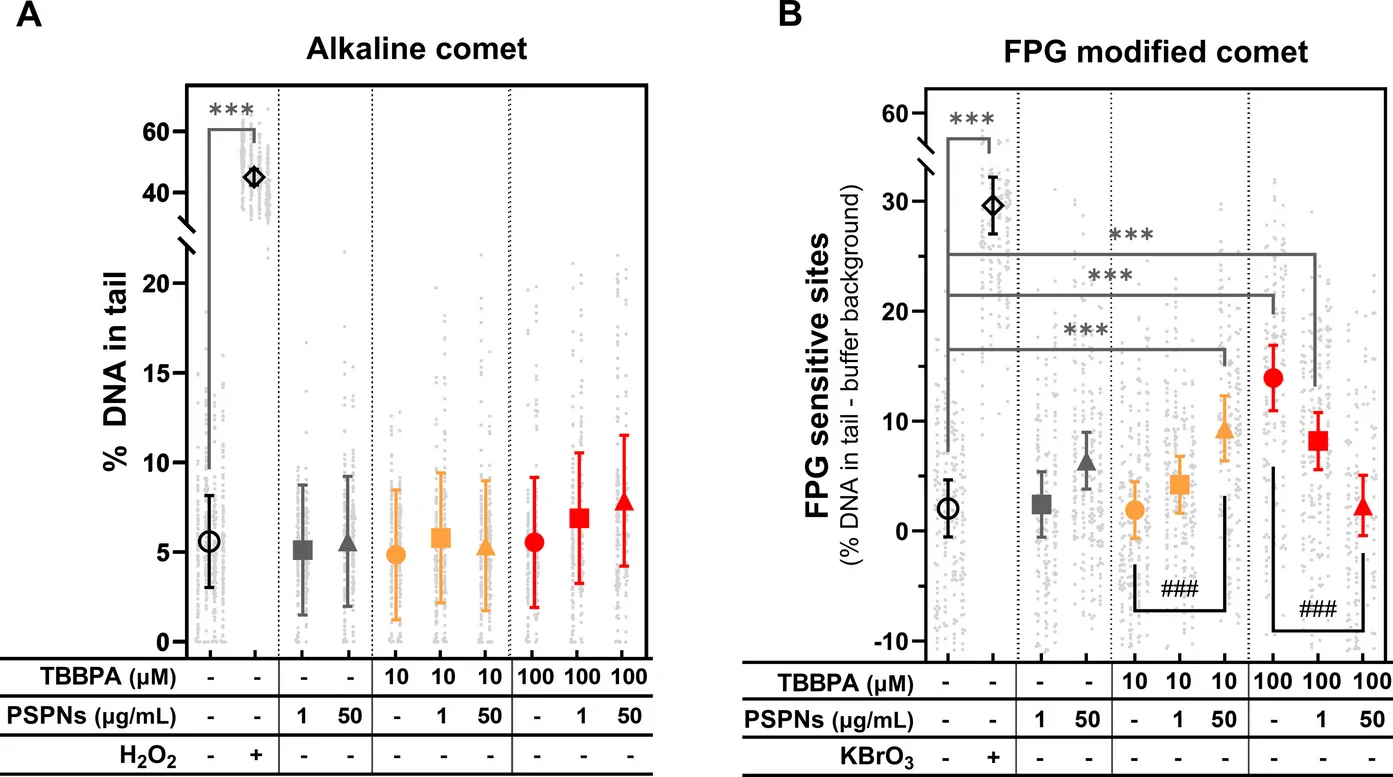
DNA damage in Caco-2/HT29-MTX co-cultures after 21 days of continuous exposure to TBBPA, PSNPs, and their combinations, assessed using the standard A and FPG-modified B comet assay. Grey dots represent individual nucleoids (50—70 per gel, only for visualization purposes and not considered for statistical analysis). Summary values (symbols) represent the mean of independent biological experiments (n = 2—3 per condition), each comprising two technical replicates (2 gels). Error bars indicate the 95% confidence interval calculated across biological (independent) experiments. Statistical analysis was performed using nested one-way ANOVA accounting for gel nested within biological experiment, followed by Sidak-adjusted pairwise comparisons (p < 0.05) among predefined groups of interest

Using the standard alkaline comet assay, we evaluated and measured direct DNA damage in the co-culture after continuous exposure to the selected TBBPA-PSNPs mixtures for 21 days (Fig. 7A). No statistically significant differences were found, neither when comparing the treatments with control untreated cells, nor when each combination was evaluated against its individual components. Nevertheless, the presence of PSNPs in combination with 100 µM TBBPA suggest a slight dose-dependent increase in the % of DNA in tail that does not reach statistical significance.

Interestingly, when the alkaline FPG-modified comet assay was performed (Fig. 7B), individual exposure to 100 µM TBBPA and the combinations of 10 µM TBBPA +50 µg/mL PSNPs and 100 µM TBBPA +1 µg/mL PSNPs showed statistically significant increases when compared with control cells. Strikingly, the presence of increasing concentrations of PSNPs led to opposite effects depending on the TBBPA condition. With 10 µM TBBPA, an increase in FPG sensitives sites was detected, although only statistically significant with the highest PSNPs concentration. On the contrary, the high amount of FPG sensitive sites detected with 100 µM TBBPA showed a decrease trend with growing PSNPs amounts, being statistically significant with the highest PSNPs condition.

**Fig. 8:**
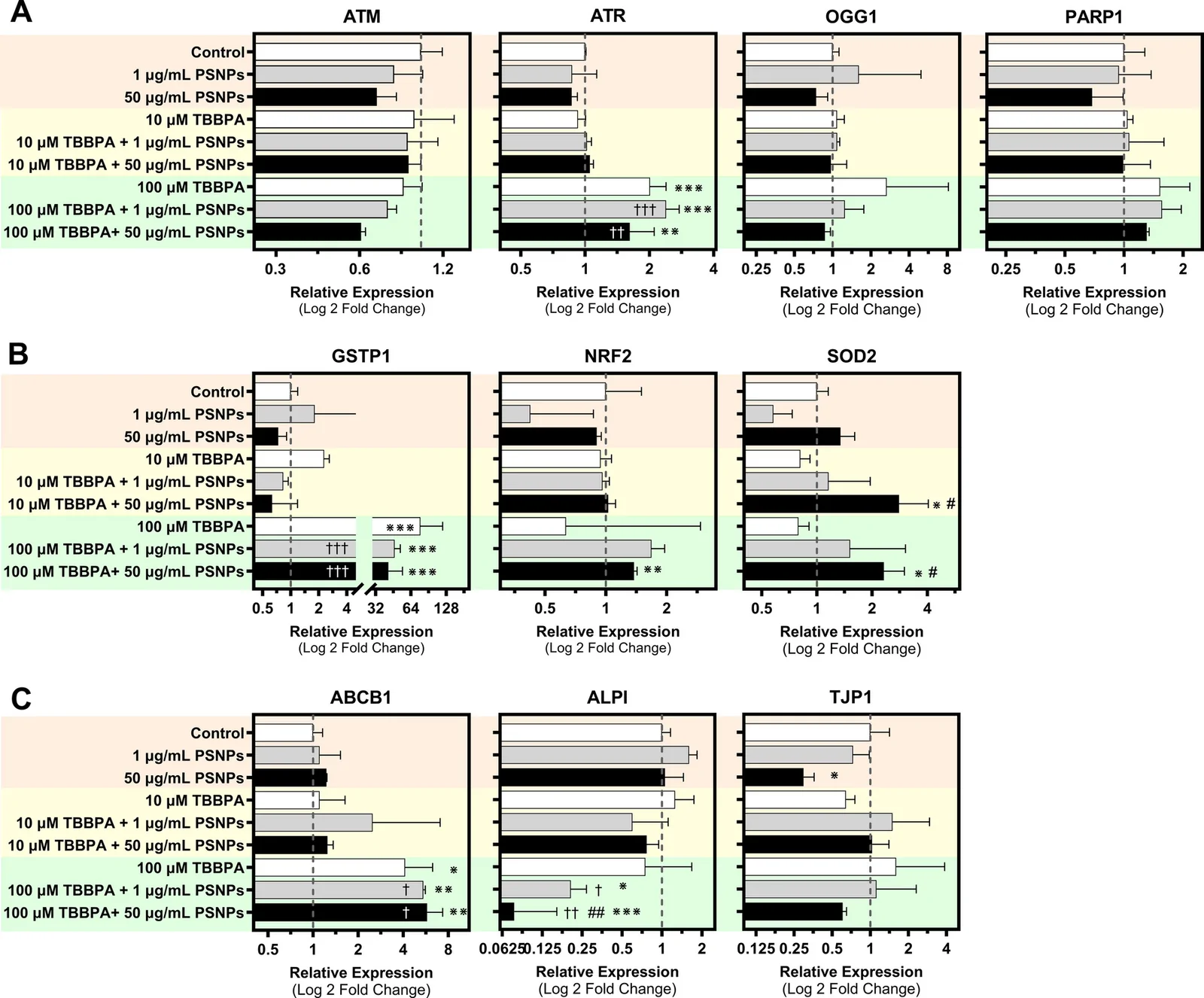
Relative expression of genes related to DNA damage response A, oxidative stress B and cell differentiation C in Caco-2/HT29-MTX co-cultures continuously exposed throughout the 21-day differentiation period. The relative expression of selected genes was evaluated by real time RT-qPCR. Bar charts show the mean ± SD of n = 3 independent biological experiments. Asterisks (*) indicate statistically significant differences compared with control; hashtags (#) indicate differences with the corresponding individual TBBPA treatment; and daggers (†) indicate differences with the corresponding individual PSNPs treatment. Determined by one-way ANOVA followed by Šídák-adjusted pairwise comparisons or Welch's ANOVA with Games-Howell (α = 0.05)

Regarding the four DNA damage-related genes evaluated (*ATM, ATR, OGG1*, and *PARP1*), we did not observe a common general response (Fig. 8A). In fact, only ATR showed statistically significant overexpression when compared with control cells with 100 µM TBBPA and both PSNPs combinations. Indeed, they were also statistically overexpressed when compared with individual PSNPs values. Neither *ATM*, *OGG1* nor *PARP1* showed relevant differences, although a tendency to downregulation was seen in *ATM* with the different 100 µM TBBPA combinations.

### Oxidative metabolism-related genes are upregulated when exposed to selected treatments

Next, we analyzed the relative expression of a selected set of oxidative stress-related genes, namely *GSTP1*,* NRF2*, and *SOD2* (Fig. 8B). A strikingly high upregulation of *GSTP1* was found following 100 µM TBBPA treatment and its combinations, that was statistically significant with control untreated cells and with individual PSNPs values. Nevertheless, no differences with individual exposure to TBBPA was found, which showed the highest overexpression values. For *NRF2* the combinations with 100 µM TBBPA tend to a slight upregulation, only statistically significant with control cells for the 50 µg/mL PSNPs combination. In the case of SOD2, the presence of PSNPs in the combination led to a dose dependent upregulation, which was statistically significant with 50 µg/mL PSNPs, both with control untreated cells and with individual exposure to TBBPA.

### Differentiation-related genes in the co-culture are altered in the presence of TBBPA and PSNPs combinations

Finally, we analyzed the relative expression of the differentiation-related genes after continuous exposure of the co-culture to TBBPA and PSNPs combinations during the differentiation process (Fig. 8C). We found variations among the studied genes. The ABC transporter gene (*ABCB1*) showed a relevant upregulation when 100 µM TBBPA was present, either alone or in combinations, that was statistically significant with control untreated cells and with individual PSNPs exposures. The microvilli-related gene *ALPI* presented the opposite behavior, with a dramatic and statistically significant dose-dependent repression detected with both 100 µM TBBPA combinations when compared with control and individual PSNPs conditions. In fact, for 100 µM TBBPA +50 µg/mL PSNPs, *ALPI* was also significantly downregulated in comparison with 100 µM TBBPA alone. In the case of *TJP1*, no clear pattern was found apart from trend towards repression with 50 µg/mL PSNPs treatments, only statistically significant for the individual exposure.

## Discussion

Given growing concerns regarding unintended exposure to contaminants of emerging concern, the present study aimed to elucidate the toxicological risks associated with joint exposure to TBBPA and PSNPs using a human intestinal Caco-2 cell model. Specifically, we investigated whether PSNPs modulate TBBPA toxicity in both differentiating and fully differentiated intestinal cells.

First, we validated our model systems by confirming the acquisition of enterocytic features, such as high TEER values (Ferruzza et al. 2012) and consistent expression of molecular the differentiation markers *ABCB1, TJP1* and *ALPI*. We observed a reduction in the expression of *ABCB1/MDR1*, which encodes the ATP-dependent efflux transporter P-glycoprotein (Zhou 2008), and *TJP1*, encoding *zonula occludens* 1 (ZO-1), a central protein in the structure of tight junctions and responsible for the integrity of the intestinal barrier (Suzuki 2012). *ABCB1* downregulation is characteristic of differentiated enterocytes with a reduced proliferation rate (Nakamura et al. 2003; Goto et al. 2003), while the reduction in *TJP1* observed in differentiated cells is consistent with the dynamic regulation of tight junction gene expression during epithelial maturation, probably due to compensatory mechanisms of other tight junction proteins (Anderson et al. 1989). On the other hand, the upregulation of *ALPI*, a gene that encodes an alkaline phosphatase associated with the development of microvilli (Matsumoto et al. 1990; Buhrke et al. 2011; Ferruzza et al. 2012), confirms the establishment of a functional brush border, a hallmark of mature enterocytes essential for nutrient absorption and xenobiotic metabolism (Buhrke et al. 2011). Overall, these markers endorsed our model using two experimental approaches (differentiation in transwell inserts, and in flat multiwell plates) as representative of differentiated human intestinal epithelium suitable for toxicity assessment.

We then exposed the differentiated Caco-2 cells to selected combinations of TBBPA and fluorescent PSNPs (fPSNPs) based on our previous studies on undifferentiated Caco-2 cells (Soto-Bielicka et al. 2024). Although the concentrations employed exceed current dietary exposure estimates for TBBPA (TDI: 0.7 µg/kg bw/day; Schrenk et al. 2024) and reported environmental levels of PSNPs, they are consistent with a hazard identification framework and allow characterisation of cellular mechanisms that may be relevant under scenarios of chronic exposure or bioaccumulation. Confocal microscopy showed incorporation and sustained intracellular accumulation of fPSNPs, accompanied by subtle structural interferences with the architecture of the intestinal barrier, with microvilli structure being the primary target. Notably, we demonstrated that fPSNPs remain inside the intestinal cells for at least 6 days post-exposure, and that the co-exposure effects on cellular adhesion structures are progressively recovered toward baseline levels following removal of contaminants. The incorporation of PSNPs has been previously reported in different intestinal model systems apparently through passive diffusion, clathrin-mediated endocytosis and/or fast endophilin mediated endocytosis (FEME) pathways as described for 26 nm PSNPs in a tri-culture small intestinal epithelial model (DeLoid et al. 2024), with smaller nanoparticles (50 *vs* 500 nm) being faster and more efficiently internalized (Liu et al. 2021; Marcellus et al. 2025). The 40 nm carboxylated PSNPs used in the present study fall within the size range associated with efficient cellular uptake in these models, which is consistent with the intracellular accumulation observed in our confocal and TEM images. Nevertheless, Marcellus et al. (2025) report a low retention time of PSNPs, with an almost complete clearance after 6 days, a finding that contrasts with the persistent intracellular retention observed in the present study up to 144 h post-exposure, and could be explained by the non-proliferative condition of our system, avoiding fPSNPs partitioning during cell division. Other long-term exposure studies to PSNPs indicate retention after 8-weeks exposure, although they were performed on Caco-2 undifferentiated cells (Domenech et al. 2021a).

The effects of PSNPs on Caco-2 structure and barrier integrity are diverse, with no clear consensus and outcomes depending on the individual or co-exposure with other contaminants and the complexity of the intestinal barrier system employed. For instance, Domenech et al. (2021b) also detected ultrastructural alterations, including changes in microvilli, after exposure to PSNPs in combination with different presentations of silver. The potential disruption of cytoskeleton by a variety of nanoparticles has been described (Ispanixtlahuatl-Meráz et al. 2018), explaining disturbances to the intestinal barrier (Cornu et al. 2020). In the present study, the less defined, broadened fluorescence signal of fPSNPs observed in cells co-exposed to TBBPA may reflect alterations in intracellular membrane organisation induced by TBBPA, which has been shown to partition extensively into phospholipid bilayers and to modify membrane fluidity and protein conformation in human erythrocyte membrane models (Jarosiewicz et al. 2021). Such membrane perturbations could alter the intracellular distribution and compartmentalisation of fPSNPs, resulting in the diffuse fluorescent pattern observed in combination treatments. To our knowledge, this is the first study to describe the retention of PSNPs in physiologically relevant models, and the recovery of cytoskeleton-related structures after withdrawal of the joint PSNPs and TBBPA exposure. Notably, Cornu et al. (2020) reported recovery of TEER values and tight junction structure after exposure to small silica nanoparticles (30 nm), suggesting that barrier restoration capacity may be size-dependent.

Given that developing intestinal epithelium may exhibit differential susceptibility to environmental toxicants compared to mature tissue, we next investigated the effects of TBBPA, PSNPs, or their combination during the active differentiation period of the intestinal barrier. To that end, we incorporated the mucus-secreting HT29-MTX cell line to the system, generating a more physiologically relevant co-culture model (Berger et al. 2017), and exposed the cells throughout the entire differentiation period (21 days). Our results after individual exposure to TBBPA and PSNPs showed cytotoxicity only for TBBPA, with similar results for both parameters evaluated, suggesting that the effects account for general cytotoxicity to the cells rather than a particular effect on metabolic activity or membrane integrity. In consequence, the combination studies showed that the main driver of the cytotoxic effects is TBBPA, and the influence of PSNPs could only be revealed with the lowest TBBPA concentration tested, similarly to what we have previously found on undifferentiated co-culture systems (Soto-Bielicka et al. 2024). Curiously, the long 21-day exposure period showed lower cytotoxic effects than our previous results on undifferentiated co-cultures during 24 h. These differences are likely explained by HT29-MTX cells modulating Caco-2 metabolic activity (Berger et al. 2017) and reduced bioavailability due to secreted mucus (Gillois et al. 2018). Indeed, higher resilience of differentiated Caco-2/HT29-MTX co-culture models has also been reported after exposure to other nanomaterials, such as silver nanoparticles (Gillois et al. 2021). Based on the available literature, no studies have investigated the combined effects of PSNPs and TBBPA in human intestinal cell models. The only other human-related combined exposure study (Liang et al. 2024) employs liver organoids and use larger microplastics (above 1 µm diameter). Their main results are related to an increased internalization of the compounds in the co-exposure and changes in transcriptomics in the organoids derived from donors with liver diseases. The remaining studies use freshwater algae (Dayana et al. 2025) and microplastics of 400 nm size, or nematodes in which intestinal damage (Chen et al. 2023) or neurodevelopmental issues (Zhao et al. 2023) were observed associated with oxidative stress.

To further investigate the potential underlying mechanisms, we employed selected endpoints to monitor DNA damage and oxidative stress responses. The generation of single- or double-strand breaks compromises the DNA structure and integrity, and their detection stands out as a primary endpoint in toxicological evaluations (Chatzidoukaki et al. 2020). Although the 100 µM TBBPA +50 µg/mL PSNPs combination had previously shown genotoxicity with the alkaline comet assay using undifferentiated co-culture of Caco-2 and HT29/MTX cells (Soto-Bielicka et al. 2024), no DNA damage was found with the new experimental settings.

We next assessed the generation of oxidative DNA injuries using the FPG modified comet assay. An unexpected response was observed, in which PSNPs modulated TBBPA-induced oxidative DNA damage in a manner dependent on the TBBPA concentration, enhancing it at the lower concentration and reducing it at the higher. This apparently incongruent situation might be explained by different circumstances. We have previously seen that an approximately 30% cytotoxicity is exerted by 100 µM TBBPA alone and in combination, a situation which may have contributed to the observed reduction on FPG sensitive sites but is unlikely to fully account for it. ATR, the primary sensor of replicative stress and single-stranded DNA (Blackford and Jackson 2017), was significantly upregulated in cells exposed to 100 μM TBBPA and all corresponding co-exposure conditions, indicating activation of the replication stress checkpoint (Cimprich and Cortez 2008). This response is consistent with the presence of SSB-induced replication fork stalling and suggests the engagement of DNA repair pathways to preserve genome integrity, thereby reducing the FPG sensitive sites detected with comet assay. On the other hand, oxidative genotoxicity was dose-dependently increased with PSNPs combined with low TBBPA (10 μM), compatible with an additive or potentiation interaction. This situation could be explained by the reported role of PSNPs as carriers of other nanomaterials, facilitating the intracellular delivery of co-occurring contaminants (Domenech et al. 2021b; Souza et al. 2022).

To elucidate the molecular response to this DNA damage, we examined expression of other key DNA damage response (DDR) genes. In contrast with *ATR, ATM*, which responds primarily to double-strand breaks (Blackford and Jackson 2017), showed no significant induction, suggesting that DSBs were unlikely to be the predominant lesions under our experimental conditions. Similarly, *OGG1*, the major DNA glycosylase responsible for the repair of oxidized guanine lesions, was not transcriptionally induced despite the clear increase in oxidative DNA damage detected by the FPG-modified comet assay. This apparent discrepancy is not unexpected, as *OGG1* activity is regulated predominantly through post-translational mechanisms, including phosphorylation, acetylation, and nuclear translocation, rather than through changes in gene expression (Boiteux and Radicella 2000). Moreover, constitutive *OGG1* expression may provide sufficient capacity to repair moderate oxidative DNA lesions without requiring detectable changes in mRNA levels.

The analysis of antioxidant genes (*GSTP1, NRF2, SOD2*) showed upregulation trends with a diversity of statistical significance support. Consistent with the activation of the DNA damage response, the most pronounced changes were also observed following exposure to TBBPA. *GSTP1*, the most strongly induced gene, was markedly upregulated following exposure to 100 μM TBBPA and remained elevated in both co-exposure groups, supporting activation of phase II detoxification mechanisms in response to oxidative stress. Likewise, *SOD2* expression increased following high-dose TBBPA exposure, with co-exposure to PSNPs further enhancing this response. This situation agrees with our previous observations in undifferentiated co-cultures (Soto-Bielicka et al. 2024), but expanding the effect to high, and not only low, TBBPA concentrations and suggesting that nanoplastics may potentiate mitochondrial antioxidant defence under oxidative stress conditions.

In contrast, *NRF2* expression was only marginally modified, which is not unexpected given that its activity is primarily regulated through post-translational modifications and nuclear translocation rather than transcriptional induction (Tonelli et al. 2018). Together with the absence of *OGG1*, these findings suggest that the antioxidant response relied on the activation of constitutively expressed factors, without requiring extensive transcriptional activation of key stress-response genes.

Among the genes associated with intestinal epithelial differentiation and barrier function, *ABCB1* was primarily induced by 100 μM TBBPA, whereas *TJP1* expression remained largely unchanged. This fact is consistent with the multifactorial regulation of ZO-1, whose activity depends predominantly on protein–protein interactions, post-translational modifications, and subcellular localization rather than on transcriptional changes (Suzuki 2012). In contrast, *ALPI* was the only differentiation marker clearly modulated by PSNPs co-exposures, which further supports our observations with TEM in fully differentiated Caco-2 cells.

Collectively, these findings point to TBBPA as the primary driver of the molecular response in the differentiating co-culture model, activating the *ATR*-dependent replication stress pathway together with phase II detoxification and mitochondrial antioxidant mechanisms. PSNPs exerted modulatory effects on this response — mainly enhancing *SOD2* expression and altering the differentiation marker *ALPI* — without substantially modifying the overall transcriptional program induced by TBBPA alone. In the fully differentiated transwell model, the progressive recovery of cellular adhesion structures following contaminant removal indicates that the intestinal epithelium retains the capacity to restore barrier integrity after transient exposure, a phenomenon consistent with adaptive repair processes described in the intestinal epithelium *in vivo*. Taken together, the two complementary models employed provide convergent evidence that TBBPA is the predominant toxicological driver in these co-exposure scenarios, while PSNPs contribute modulatory effects whose biological significance may increase under conditions of sustained intracellular accumulation.

Nevertheless, the prolonged intracellular persistence of PSNPs within intestinal epithelial cells suggests that even these modest modulatory effects could become biologically relevant under repeated or continuous exposure scenarios. Sustained intracellular accumulation may enhance or prolong cellular responses to co-occurring environmental contaminants, underscoring the importance of testing multiple dose levels and the complexity of nanomaterial-contaminant interactions in risk assessment.

## Supplementary Information

Below is the link to the electronic supplementary material.

Supplementary file1 (DOCX 562 KB)

Supplementary file2 (DOCX 56 KB)

Supplementary file3 (DOCX 3849 KB)

Supplementary file4 (DOCX 358 KB)

Supplementary file5 (DOCX 19 KB)

Supplementary file6 (DOCX 21 KB)

Supplementary file7 (DOCX 16 KB)

## Data Availability

The raw data supporting the findings of this study are publicly available in the e-cienciaDatos repository (UAM) at https://doi.org/10.21950/HWW3QO. Soto Bielicka, Patricia; Sanz Alferez, Soledad; Hazen De San Juan, M. Jose; Fernández Freire, Paloma; Peropadre, Ana, 2026, "Co-exposure to polystyrene nanoparticles and tetrabromobisphenol A induces genotoxicity and disrupts intestinal barrier differentiation", https://doi.org/10.21950/HWW3QO, e-cienciaDatos, V2.
