# Supplementary material for: Co-exposure to polystyrene nanoparticles and tetrabromobisphenol A induces genotoxicity and interferes with intestinal barrier differentiation
Source: Cell Biol Toxicol. 2026 Jul 10;42(1):113. doi: 10.1007/s10565-026-10228-6 (PMC13638729; doi:10.1007/s10565-026-10228-6)
Supplement: Supplementary file 1 [file 10565_2026_10228_MOESM1_ESM.docx]

**Co-exposure to polystyrene nanoparticles and tetrabromobisphenol A induces genotoxicity and interferes with intestinal barrier differentiation.**

Patricia Soto-Bielicka^1^, Soledad Sanz-Alférez^1^, M. José Hazen^1^, Paloma Fernández Freire^1^*, Ana Peropadre^1^

^1^ Biology Department, Faculty of Sciences, Autonomous University of Madrid, Madrid, 28049, Spain.

**Supp. Figure 1. Schematic overview of the experimental workflow**. Top, Caco-2 cells were seeded on transwell inserts, differentiated for 21 days, and exposed to TBBPA and/or PSNPs for 24 h. Thereafter, the inserts were fixed at different time points (24 h to evaluate intestinal barrier integrity, or 2–144 h to evaluate post-exposure recovery) prior to TEM ultrastructural analysis and confocal laser scanning microscopy imaging. Bottom, Caco-2/HT29-MTX co-cultures (9:1) were seeded in 6-well or 96-well plates and exposed to TBBPA and/or PSNPs for up to 21 days. Then, different assays were performed to evaluate genotoxicity (comet assay), gene expression (Real Time RT-qPCR), and cytotoxicity assessment (AB/CFDA-AM).
