# Supplementary material for: Co-exposure to polystyrene nanoparticles and tetrabromobisphenol A induces genotoxicity and interferes with intestinal barrier differentiation
Source: Cell Biol Toxicol. 2026 Jul 10;42(1):113. doi: 10.1007/s10565-026-10228-6 (PMC13638729; doi:10.1007/s10565-026-10228-6)
Supplement: Supplementary file 2 [file 10565_2026_10228_MOESM2_ESM.docx]

**Co-exposure to polystyrene nanoparticles and tetrabromobisphenol A induces genotoxicity and interferes with intestinal barrier differentiation.**

Patricia Soto-Bielicka^1^, Soledad Sanz-Alférez^1^, M. José Hazen^1^, Paloma Fernández Freire^1^*, Ana Peropadre^1^

^1^ Biology Department, Faculty of Sciences, Autonomous University of Madrid, Madrid, 28049, Spain.

**Supp. Figure 2. TEER values after 24 h exposure to different combinations of TBBPA and PSNPs on a Caco-2 derived intestinal barrier.** Caco-2 cells were grown on transwell inserts and allowed to differentiate (DF) for 21 days. Afterwards, they were exposed to selected combinations of TBBPA and PSNPs during 24 h prior to TEER measurements.
