# Supplementary material for: Co-exposure to polystyrene nanoparticles and tetrabromobisphenol A induces genotoxicity and interferes with intestinal barrier differentiation
Source: Cell Biol Toxicol. 2026 Jul 10;42(1):113. doi: 10.1007/s10565-026-10228-6 (PMC13638729; doi:10.1007/s10565-026-10228-6)
Supplement: Supplementary file 3 [file 10565_2026_10228_MOESM3_ESM.docx]

**Co-exposure to polystyrene nanoparticles and tetrabromobisphenol A induces genotoxicity and interferes with intestinal barrier differentiation.**

Patricia Soto-Bielicka^1^, Soledad Sanz-Alférez^1^, M. José Hazen^1^, Paloma Fernández Freire^1^*, Ana Peropadre^1^

^1^ Biology Department, Faculty of Sciences, Autonomous University of Madrid, Madrid, 28049, Spain.

**Supp. Figure 3. Individual cytotoxicity of Caco-2 after continuous exposure during the differentiation process either to TBBPA (A) or PSNPs (B).** Scatter plots of the metabolic activity (AB) and plasma membrane integrity (CFDA-AM), showing dots for each biological experiment (n = 3 for TBBPA and 2 for PSNPs, with 6 technical replicates each) and a line for their mean. Values are expressed as percentage of control untreated cells (%C). Asterisks indicate statistically significant differences compared to control cells, with p < 0.05 (*), p < 0.01 (**) or p < 0.0001 (****), after one-way ANOVA with Dunnett’s T3 tests (AB) or Kruskal-Wallis with Dunn’s test (CFDA-AM) for TBBPA, and Kruskal-Wallis with Dunn’s test (AB) or ordinary one-way ANOVA with Dunnett’s T3 tests (CFDA-AM) for PSNPs.
