# Supplementary material for: Co-exposure to polystyrene nanoparticles and tetrabromobisphenol A induces genotoxicity and interferes with intestinal barrier differentiation
Source: Cell Biol Toxicol. 2026 Jul 10;42(1):113. doi: 10.1007/s10565-026-10228-6 (PMC13638729; doi:10.1007/s10565-026-10228-6)
Supplement: Supplementary file 4 [file 10565_2026_10228_MOESM4_ESM.docx]

**Co-exposure to polystyrene nanoparticles and tetrabromobisphenol A induces genotoxicity and interferes with intestinal barrier differentiation.**

Patricia Soto-Bielicka^1^, Soledad Sanz-Alférez^1^, M. José Hazen^1^, Paloma Fernández Freire^1^*, Ana Peropadre^1^

^1^ Biology Department, Faculty of Sciences, Autonomous University of Madrid, Madrid, 28049, Spain.

**Supp. Figure 2.** **PROAST dose–response modelling of TBBPA–PSNPs mixtures.** Dose-addition models were fitted separately for the Alamar Blue (AB, top plots) and carboxyfluorescein diacetate acetoxymethyl ester (CFDA-AM, bottom plots) endpoints. Consistency of the mixture data with the dose-addition assumption was evaluated using the log-likelihood ratio test (loglik; p > 0.05 indicating no significant deviation from dose addition). Model selection was based on the Akaike Information Criterion (AIC), and the model with the lowest AIC was used to estimate the critical effect dose (CED) at a critical effect size (CES, equivalent to BMR) of 5%, together with its lower and upper confidence bounds (CEDL and CEDU; corresponding to BMD, BMDL and BMDU, respectively). TBBPA was selected as the reference compound, and mixture doses were expressed as TBBPA-equivalent doses. The relative potency factor (RPF) of PSNPs was estimated relative to TBBPA.
