# Supplementary material for: Co-exposure to polystyrene nanoparticles and tetrabromobisphenol A induces genotoxicity and interferes with intestinal barrier differentiation
Source: Cell Biol Toxicol. 2026 Jul 10;42(1):113. doi: 10.1007/s10565-026-10228-6 (PMC13638729; doi:10.1007/s10565-026-10228-6)
Supplement: Supplementary file 5 [file 10565_2026_10228_MOESM5_ESM.docx]

**Co-exposure to polystyrene nanoparticles and tetrabromobisphenol A induces genotoxicity and interferes with intestinal barrier differentiation.**

Patricia Soto-Bielicka^1^, Soledad Sanz-Alférez^1^, M. José Hazen^1^, Paloma Fernández Freire^1^*, Ana Peropadre^1^

^1^ Biology Department, Faculty of Sciences, Autonomous University of Madrid, Madrid, 28049, Spain.

**Supp. Table 1.** Primers used for Real Time RT-qPCR analysis.

| Pathway | Gene | Primers |
| --- | --- | --- |
| DNA damage | OGG1 | 5' - GAGCCCCCAGACCAACAAGG -3' |
|  |  | 5' - TGGGGTCCGAGAAAGGTCAT - 3' |
|  | ATM | 5' - GGACAGTGGAGGCACAAAAT - 3' |
|  |  | 5' - GTGTCGAAGACAGCTGGTGA - 3' |
|  | ATR | 5' - CTCGCTGAACTGTACGTGGA - 3' |
|  |  | 5' - GCATAGCTCGACCATGGATT - 3' |
|  | PARP1 | 5' - AAGGCGAATGCCAGCGTTAC - 3' |
|  |  | 5' - GCACTCTTGGAGACCATGTCA - 3' |
| Oxidative stress | GSTP1 | 5' - TGGACATGGTGAATGACGGCGT - 3' |
|  |  | 5' - GGTCTCAAAAGGCTTCAGTTGCC - 3' |
|  | NRF2 | 5' - CAGTCAGCGACGGAAAGAGT - 3' |
|  |  | 5' - ACCTGGGAGTAGTTGGCAGA - 3' |
|  | SOD2 | 5' - CTGGACAAACCTCAGCCCTAAC - 3' |
|  |  | 5' - AACCTGAGCCTTGGACACCAAC - 3' |
| Diferentiation | ALPI | 5’ - GCTCAGGACAGCAAAGCCTA - 3’ |
|  |  | 5’ - AGGTGGGGAGCAGGATAACT - 3’ |
|  | MDR1 | 5’ - GTAGCGGCTCTTCCAAGCTC - 3’ |
|  |  | 5’ - AGGGTTAGCTTCCAACCACG - 3’ |
|  | TJP1 | 5’ - ACCAGAAATACCTGACGGTGC - 3’ |
|  |  | 5’ - AGGGTTTTCCTTGGCTGACA - 3’ |
| *Housekeeping* | 18S | 5' - AACGTCTGCCCTATCAACTTT - 3' |
|  |  | 5' - TGGATGTGGTAGCCGTTTTCT - 3' |
|  | CycloB | 5' - GCAAGATCGAGGTGGAGAAG - 3' |
|  |  | 5' - CTGTGGAATGTGAGGGGAGT - 3' |
