# Supplementary material for: Co-exposure to polystyrene nanoparticles and tetrabromobisphenol A induces genotoxicity and interferes with intestinal barrier differentiation
Source: Cell Biol Toxicol. 2026 Jul 10;42(1):113. doi: 10.1007/s10565-026-10228-6 (PMC13638729; doi:10.1007/s10565-026-10228-6)
Supplement: Supplementary file 6 [file 10565_2026_10228_MOESM6_ESM.docx]

**Co-exposure to polystyrene nanoparticles and tetrabromobisphenol A induces genotoxicity and interferes with intestinal barrier differentiation.**

Patricia Soto-Bielicka^1^, Soledad Sanz-Alférez^1^, M. José Hazen^1^, Paloma Fernández Freire^1^*, Ana Peropadre^1^

^1^ Biology Department, Faculty of Sciences, Autonomous University of Madrid, Madrid, 28049, Spain.

**Supp. Table 2.** Benchmark doses (BMD) in µM for Caco-2 and the co-culture after 21 days of continuous exposure to TBBPA.

|  | Caco-2 | | Caco-2/HT29-MTX | |
| --- | --- | --- | --- | --- |
|  | BMDL | BMDU | BMDL | BMDU |
| AB | 52.5 | 71.5 | 76.9 | 100 |
| CFDA-AM | 49.9 | 69.7 | 96.7 | - |
