# Supplementary material for: Co-exposure to polystyrene nanoparticles and tetrabromobisphenol A induces genotoxicity and interferes with intestinal barrier differentiation
Source: Cell Biol Toxicol. 2026 Jul 10;42(1):113. doi: 10.1007/s10565-026-10228-6 (PMC13638729; doi:10.1007/s10565-026-10228-6)
Supplement: Supplementary file 7 [file 10565_2026_10228_MOESM7_ESM.docx]

**Co-exposure to polystyrene nanoparticles and tetrabromobisphenol A induces genotoxicity and interferes with intestinal barrier differentiation.**

Patricia Soto-Bielicka^1^, Soledad Sanz-Alférez^1^, M. José Hazen^1^, Paloma Fernández Freire^1^*, Ana Peropadre^1^

^1^ Biology Department, Faculty of Sciences, Autonomous University of Madrid, Madrid, 28049, Spain.

**Supp. Table 3.** PROAST-derived BMD analysis for the co-culture after 21 days of exposure to the TBBPA-PSNPs combinations.

|  |  | BMDL | BMDU |
| --- | --- | --- | --- |
| AB | TBBPA (CED) | 62.50 | 79.20 |
|  | PSNPs (RPF) | 0.12 | 0.46 |
| CFDA-AM | TBBPA (CED) | 60.80 | 78.70 |
|  | PSNPs (RPF) | 0.00 | 0.13 |
| *CED: Critic effective dose (µM)*  *RPF: Relative potency factor* | |  |  |
